# Supplementary material for: Modelling the effects of the repellent scent marks of pollinators on their foraging efficiency and the plant-pollinator community
Source: PLoS One. 2021 Sep 8;16(9):e0256929. doi: 10.1371/journal.pone.0256929 (PMC8425561; doi:10.1371/journal.pone.0256929)
Supplement: S1 File — Model description is based on the ODD (Overview, Design concepts, Details) protocol. (DOCX) [file pone.0256929.s001.docx]

S1 File – Detailed model description

We developed an agent-based model to examine how the use of scent marks as repellent public information will affect the foraging of pollinators. The model description is based on the ODD (Overview, Design concepts, Details) protocol [1,2]. The model is coded in Python 3.8 [3] and uses *numpy* [4], *scipy* [5], and *pandas* [6] packages. Part of the code is based on the structure of the *Py_IBM* package [7]. The code and its requirements are available on gitlab. The analyses were performed with R 4.0.3 [8] using the *Rmisc* [9] and *plyr* [10] packages. The graphs were created with the *ggplot2* package [11].

All the parameters can be modified by the user. The results were exported from the simulation.

1. Purpose

Our model was designed to simulate the foraging journey of pollinators in a meadow under different competition pressures. We aim to compare the amount of nectar collected by pollinators according to their strategy (using or ignoring scent marks).

2. Entities, state variables, and scales

The pollinators forage in an unknown environment (i.e., the meadow) with a user-specified flower density.

## 2.1 Environment

The meadow is a continuous torus in 2D. Due to the random distribution and stochasticity of the model, the possibility that two individuals of the same species (i.e., flowers or pollinators) are simultaneously located in the same place is insignificant. The individual’s size is decided by the precision of the software. To enforce this, multiple pollinators cannot visit the same flower at the same time. The environment is characterised by two static variables that do not change during the simulation:

- time spent by the pollinator in the flower during the visit (*cost*);
- continuous square area of a user-specified size (*topology*).

The environment keeps track of the discrete time during the simulation with the dynamic variable *step*. The values of all the parameters and variables are indicated in Table S1.1.

Table S1.1: Overview of parameters, variables, and default values for the environment/world

| ***Parameters*** | **Value** |
| --- | --- |
| Cost: *positive integer* | 5 |
| Number of steps that the pollinator remains in a flower after visiting it |  |
| Topology: *landscape instance (size_map)* | 50 |
| Continuous square area object on which agents live (size_map*size_map) |  |
| ***Variables*** | **Initialisation** |
| Step: *integer* | 0 |
| Temporal state of the simulation |  |

## 2.2 Flowers

The flowers are immobile agents characterised by four static variables (*SV*) and six dynamic variables:

- speed of nectar refill, identical for all flowers (*coeff_nectar*) (*SV*);
- speed of the disappearance of the scent mark left by the pollinator. In our present analysis, we chose to make this mark a reliable signal of nectar refill (*coeff_mark*) (*SV*);
- unique agent serial number of the flower (*ID*) (*SV*);
- position of the flower on the 2D map (*position*) (*SV*);
- nectar quantity inside the flower at each step of the simulation (*nectar_quantity*);
- concentration of the scent mark on the corolla at each step of the simulation (*scent_mark*);
- variable indicating if a pollinator is above the flower; impossible for two pollinators to be present simultaneously (*pollinator_above*);
- variable indicating if a pollinator is inside the flower; impossible for two pollinators to be present simultaneously (*pollinator_inside*);
- time since the last visit of a pollinator, update at each step (*step_no_visit*);
- total number of pollinator visits (*nb_visits*).

The values of all the parameters and variables are indicated in Table S1.2.

Table S1.2: Overview of parameters, variables, and default values for the flower agent

| ***Parameters*** | **Value** |
| --- | --- |
| Coeff_nectar: *float or integer*  Coefficient of the linear function for the nectar evolution | [0.001, 0.0025, 0.005] |
| Coeff_mark: *float or integer*  Coefficient of the linear function for the scent mark loss | coeff_nectar |
| ***Variables*** | **Initialisation** |
| ID: *integer*  Agent serial number (identical throughout the simulation) | In order (by model) |
| Position: *tuple*  (x, y) coordinate pair for the agent’s current position | Randomly (by model) |
| Nectar_quantity: *positive float or null*  Quantity of nectar in the flower; initially 1 (full) | 1 |
| Scent_mark: *positive float or null*  Concentration of scent_mark on the corolla; initially 0 (no scent_mark). | 0 |
| Pollinator_above: *integer or none*  If a pollinator is above the flower, the pollinator’s serial number, else None | None |
| Pollinator_inside: *integer or none*  If a pollinator is inside the flower, the pollinator’s serial number, else None | None |
| Step_no_visit: *positive integer or null*  Number of steps since the last visit | -1 |
| Nb_visits: *Integer*  Number of pollinator visits during the simulation | 0 |

## 2.3 Pollinators

Pollinators are mobile agents characterised by four static variables (*SV*) and seven dynamic variables:

- use of the scent mark (*detect_mark*). In any individual simulation, all pollinators are identical regarding this behavioural trait (using or ignoring scent marks) (SV);
- flower detection radius around the pollinator, identical for all pollinators (*view_radius*) (*SV*);
- degree of inertia in the pollinator’s movements (more details provided below in section 3.2 on the process scheduling of movement) (*sigma*) (*SV*);
- unique agent serial number of the pollinator (*ID*) (*SV*);
- position of the pollinator on the 2D map at each step of the simulation (*position*);
- previous position of the pollinator on the 2D map at each step of the simulation (*previous_position*);
- total amount of nectar collected during the simulation, updated at each step (*nectar_quantity*);
- if the pollinator is in a flower, the number of *steps* since arriving (*step_in_flower*);
- number of times the pollinator takes nectar (*times_of_nectar_taken*);
- variable indicating if the pollinator is above or in a flower, and if so, which one (*on_flower*);
- list of every flower visited given that the pollinator cannot visit the same flower twice (more details provided below in section 3.2 on the process scheduling of movement) (*already_detected_flowers*).

The values of all the parameters and variables are indicated in Table S1.3.

Table S1.3: Overview of parameters, variables, and default values for the pollinator agent

| ***Parameters*** | **Value** |
| --- | --- |
| Detect_mark: *bool*  Pollinator’s ability to use scent marks | True or False |
| View_radius: *positive float or integer*  Radius of the pollinator’s circle view (i.e., radius in which flowers are detected) | 1 |
| Sigma_mvt: *positive float*  Standard deviation of the normal law of movement | 0.1 |
| ***Variables*** | **Initialisation** |
| ID: *integer*  Agent’s serial number (identical throughout the simulation) | In order (by model) |
| Position: *tuple*  (x, y) coordinate pair for the agent’s current position | Randomly (by model) |
| Previous_position: *tuple or none*  (x, y) coordinate pair for the agent’s last position | None |
| Nectar_quantity: *positive float or null*  Quantity of nectar collected by the pollinator; initially null | 0 |
| On_flower: *integer or none*  If the individual is on or in a flower, the flower’s serial number, else None | None |
| Step_in_flower: *positive integer or null*  Numbers of steps since the pollinator arrived in the flower | 0 |
| Times_of_nectar_taken: *positive integer or null*  Numbers of times that the individual took nectar | 0 |
| Already_detected_flowers: *list*  List of flower IDs visited during the foraging journey | Empty list |

# 3. Process overview and scheduling

## 3.1 Temporal scales and processes

We use a discrete model. One step represents the time needed for one action to be completed by the pollinators. The entire simulation (1,000 steps) mimics the foraging journey of a pollinator. Due to the configuration of the agent-based model, the pollinators move one after the other. The order in which the pollinators take action is randomly defined at the beginning of each step.

## 3.2 Pollinator process scheduling

The graphical depiction of these processes is illustrated in Fig S1.1.


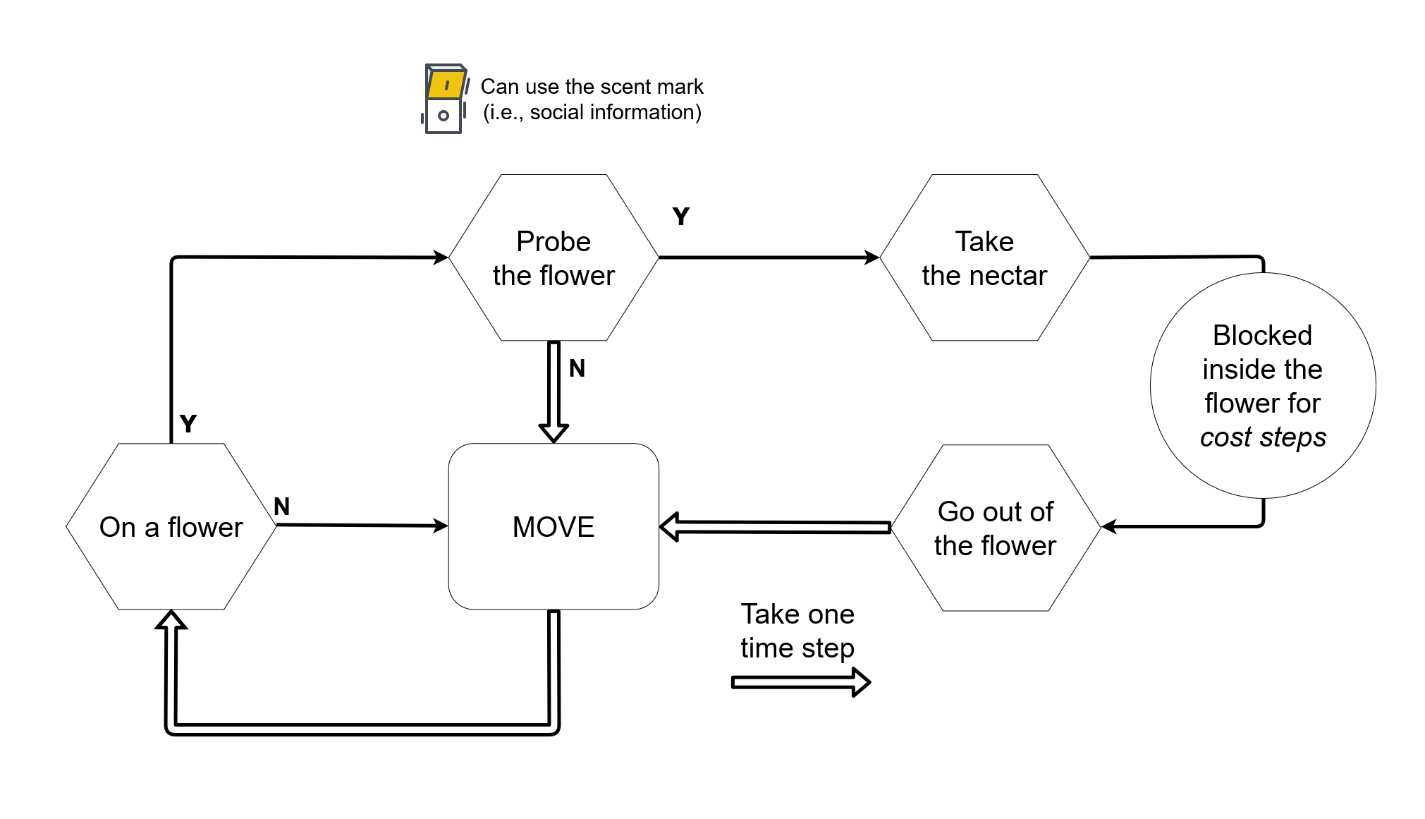


Fig S1.1: Scheduling of the pollinator

**PROBE THE FLOWER:** When the pollinator is on a flower, its behaviour depends on whether it uses the scent mark or not. If the pollinator uses this source of public information (i.e., scent mark), the probability of visiting the flower is the outcome of a Bernoulli trial with the probability set to correspond to [1-scent mark of the flower]. In the case of success, the pollinator visits the flower and takes the nectar, otherwise it will move at the next step. If the pollinator does not use the scent mark, it will visit the flower regardless of the presence of nectar. Whether or not the flower is visited, it is added to the *already_detected_flowers* list of pollinators and the value of *pollinator_above* is set to *None*. If the pollinator visits the flower, the *pollinator_inside* variable is updated with the ID of the pollinator.

**TAKE THE NECTAR:** When a pollinator visits the flower, the *pollinator_inside* variable of the flower is updated with the ID of the pollinator. When a pollinator enters a flower, it takes all the available nectar, and this quantity is added to the total amount of nectar collected. The nectar quantity of the flower is set to 0. The pollinator variables *step_in_flower* and *times_of_nectar_taken*, as well as the flower variable *nb_visits* are all updated by adding 1.

**BLOCKED IN THE FLOWER:** Until the *step_in_flower* is equal to *cost*, the pollinator is blocked in the flower. At every step, this variable increases by 1.

**GO OUT OF THE FLOWER:** When the *step_in_flower* variable is equal to the *cost* at the beginning of the turn, the pollinator moves. The values of the pollinator variables *step_in_flower* and *on_flower*, as well as the flower variables *pollinator_inside* and *step_no_visit* are set to 0 or None.

**MOVE:** The graphical description of the move function is shown in Fig S1.2. The function *move_or_not* is used after sorting the pollinators. If the pollinator is not in a flower, it will move. If there is a new free flower (i.e., not in the *already_detected_flowers* list, with no pollinator above or inside) within its detection radius, the pollinator moves to it. If there is more than one flower, a random draw is made to select the flower. If there is no free flower within the detection radius, it will move in a direction that departs from the previous direction by an angle taken from a Gaussian law centred on 0, with a standard error of *sigma*. This approaches a correlated random walk [12,13] and has been used previously in foraging ABM models [14,15]. The pollinator moves a distance of *view_radius* in the new direction. The previous and current positions are updated.


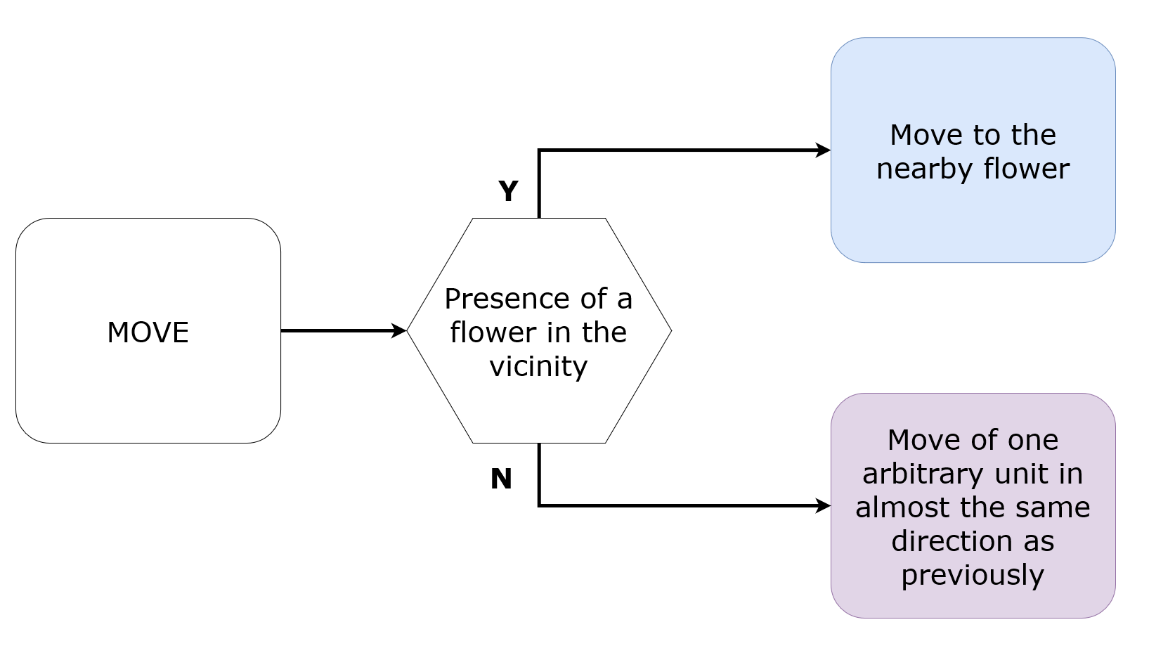


Fig S1.2: Details of the move function

## 3.3 Flower process scheduling

When all the pollinators have completed their actions, the scent mark and nectar quantity of every flower are updated (in the order of their ID). The graphical description of these processes is shown in Fig S1.3.


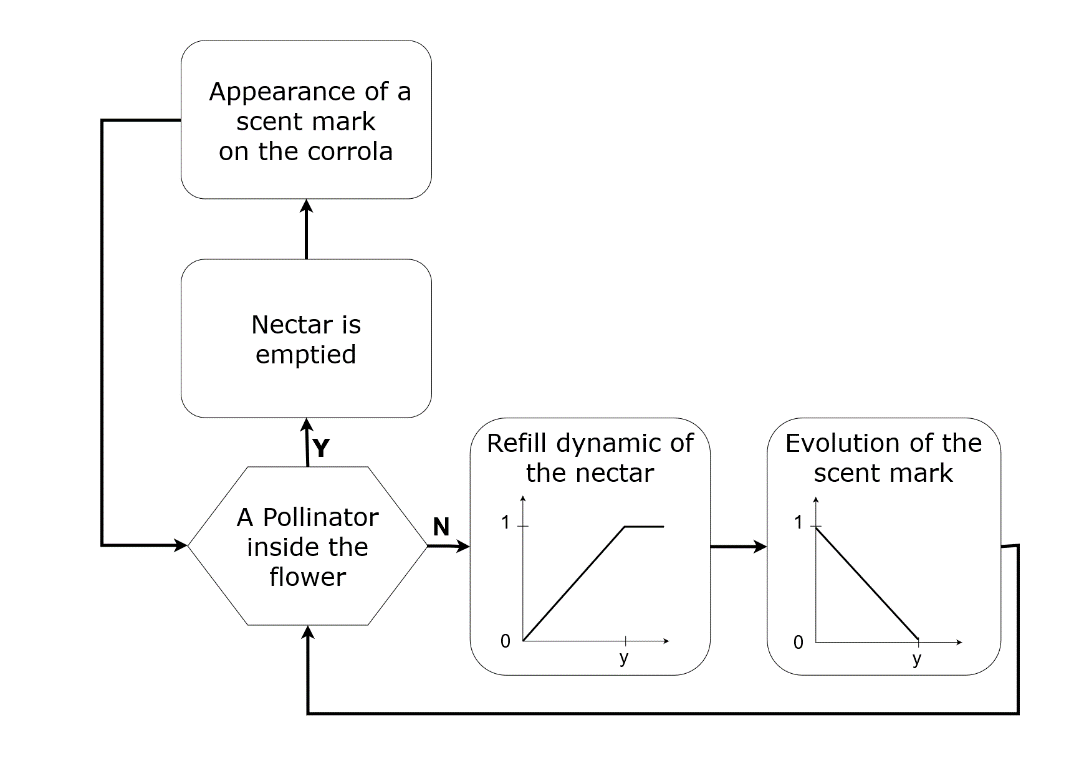


Fig S1.3: Scheduling of the flower

**UPDATE NECTAR QUANTITY:** If there is a pollinator inside the flower, the nectar quantity will be set to 0. Otherwise, the nectar quantity is updated with the formula: *Step_no_visit * coeff_nectar*. If the result is greater than 1, the value is set to 1.

**UPDATE SCENT MARK CONCENTRATION:** If there is a pollinator inside the flower, the scent mark will be set to 1. Otherwise, the scent mark is updated with the formula: 1 + *Step_no_visit * coeff _nectar*. If the result is less than 0, the value is set to 0.

# 4. Design concepts

## 4.1 Basic principles

The model follows the foraging journey of pollinators in a stable environment. The pollinator faces an unknown environment and is in competition with fellow pollinators. Its capacity to use different types of information will change its journey and relative success.

## 4.2 Emergence

At the end of each simulation, we extract the following values:

- mean nectar quantity collected by pollinators during the simulation (+ coefficient of variation);
- mean nectar quantity in the flower at the end of the simulation (+ coefficient of variation);
- mean number of visits per flower during the simulation (+ coefficient of variation).

## 4.3 Stochasticity

Several events in our model are stochastic. The flowers and pollinators are randomly placed on the map during the initialisation. At each time step, pollinators can diverge from the previous direction according to a Gaussian law. When multiple flowers are present in the pollinator’s detection radius, the choice of the flower to visit is random. Finally, when pollinators use the scent mark, the final decision about whether to probe the flower is a Bernoulli trial whose probability is negatively correlated to the intensity of the scent mark.

## 4.4 Observations

Multiple simulations were run in parallel, and all the data were saved in a single table, with each line corresponding to one simulation. In addition to the user-specified parameters and the emergent variables mentioned above, the duration of the simulation and the number of unvisited flowers were extracted.

# 5. Initialisation

All the parameter values used during our study are provided in Table S.4. At the beginning of the simulation, the following values were created:

- world, a toroid of *size_map²*;
- number of flowers calculated to ensure a user-defined flower density [number of flowers = *(flower_density * size_map ** 2) / 100*]*.* These flowers are randomly positioned.
- number of pollinators calculated to ensure a user-defined ratio of pollinators per flower [number of pollinators = (*prop_pollinator * nb_flowers) / 100*]. These pollinators are randomly placed on the map.

At the beginning of the simulation, the flowers are full of nectar (1), and the pollinators do not have any (0).

Table S.4: List of the model parameters and their variation ranges. The values exploited during sensitivity analysis appear in the “interval”, while the column “value” presents values exploited in the simulation experiments and the default values for the remaining parameters.

| ***Parameters*** | **Value** | **Interval (sensitivity)** |
| --- | --- | --- |
| Size_map: *positive integer*  One side of the square for the map | 50 | [30 – 100] |
| Flowers_density: *positive integer*  Number of flowers desired on the map, depending on the area of the map (in percent) | 10% | [2% – 20%] |
| Proportion_pollinators: *positive integer*  Number of pollinators desired on the map, depending on the number of flowers (in percent) | Interest variable  [10% – 200%] | [10% – 200%] |
| Ind_info: *bool*  Define whether or not the pollinator can use the scent mark | True or False | True or False |
| View_radius: *positive float or integer*  Radius of the pollinator’s view (less than half of size_map) | 1 | 1 |
| Sigma_mvt: *positive float*  Value for the normal law to define the randomness of movement | 0.1 | [0.01 – 0.4] |
| Cost: *positive integer*  Number of steps during which the pollinator remains in the flower to take the nectar | 5 | [1 – 10] |
| Coeff_nectar: *positive float or integer*  Coefficient of the linear function for the nectar evolution | [0.001, 0.0025, 0.005] | [0.001 – 0.005] |
| Coeff_mark: *positive float or integer*  Coefficient of the linear function for the scent mark loss | - coeff_nectar | - coeff_nectar |
| Nb_step: *positive integer*  Number of steps in the simulation | 1000 | [700 – 1300] |

# 6. References

1. Grimm V, Berger U, DeAngelis DL, Polhill JG, Giske J, Railsback SF. The ODD protocol: A review and first update. Ecol Model. 2010;221(23):2760–8.

2. Grimm V, Railsback SF, Vincenot CE, Berger U, Gallagher C, DeAngelis DL, et al. The ODD Protocol for Describing Agent-Based and Other Simulation Models: A Second Update to Improve Clarity, Replication, and Structural Realism. J Artif Soc Soc Simul. 2020;23(2):7.

3. Python Software Foundation. Python Language Reference, version 3.8, Available at http://www.python.org. 2019.

4. Harris CR, Millman KJ, van der Walt SJ, Gommers R, Virtanen P, Cournapeau D, et al. Array programming with NumPy. Nature. 2020;585(7825):357–62.

5. SciPy 1.0 Contributors, Virtanen P, Gommers R, Oliphant TE, Haberland M, Reddy T, et al. SciPy 1.0: fundamental algorithms for scientific computing in Python. Nat Methods. 2020;17(3):261–72.

6. McKinney W. Data Structures for Statistical Computing in Python. In Austin, Texas; 2010 [cited 2021 Feb 25]. p. 56–61. Available from: https://conference.scipy.org/proceedings/scipy2010/mckinney.html

7. Frazao F. Py_IBM [Internet]. 2018. Available from: https://github.com/fsfrazao/Py_IBM

8. R Core Team. R: A language and environment for statistical computing [Internet]. Vienna, Austria; 2020. Available from: https://www.R-project.org/

9. Hope RM. Rmisc: Rmisc: Ryan Miscellaneous. [Internet]. 2013. (R package). Available from: https://CRAN.R-project.org/package=Rmisc

10. Wickham H. The Split-Apply-Combine Strategy for Data Analysis. J Stat Softw [Internet]. 2011 [cited 2021 Mar 1];40(1). Available from: http://www.jstatsoft.org/v40/i01/

11. Wickham H, Chang W, Henry L, Pedersen T, Takahashi K, Wilke C, et al. ggplot2: Elegant Graphics for Data Analysis. Springer-Verl N Y. 2016;

12. Bovet P, Benhamou S. Spatial analysis of animals’ movements using a correlated random walk model. J Theor Biol. 1988 Apr;131(4):419–33.

13. Viswanathan GM, Buldyrev SV, Havlin S, da Luz MGE, Raposo EP, Stanley HE. Optimizing the success of random searches. Nature. 1999 Oct;401(6756):911–4.

14. Deygout C, Gault A, Sarrazin F, Bessa-Gomes C. Modeling the impact of feeding stations on vulture scavenging service efficiency. Ecol Model. 2009 Aug;220(15):1826–35.

15. Deygout C, Gault A, Duriez O, Sarrazin F, Bessa-Gomes C. Impact of food predictability on social facilitation by foraging scavengers. Behav Ecol. 2010;21(6):1131–9.
