# Supplementary material for: Modelling the effects of the repellent scent marks of pollinators on their foraging efficiency and the plant-pollinator community
Source: PLoS One. 2021 Sep 8;16(9):e0256929. doi: 10.1371/journal.pone.0256929 (PMC8425561; doi:10.1371/journal.pone.0256929)
Supplement: S2 File — Sensitivity analysis. (DOCX) [file pone.0256929.s002.docx]

S2 File – Sensitivity analysis

The impact of the model parameters on the quantity of nectar collected by pollinators was examined in the simulation analysis for a range of parameters (Table S2.1). We ran 10,000 simulations, and for each simulation, a random value in the interval was assigned to the parameter. Their impact was studied with a random forest analysis in R version 4.0.3 [1] and the *randomForest* package [2]. We built 2,000 regression trees, and three variables were tried at each split.

Table S2.1: List of model parameters assessed during the sensitivity analysis and the variation range examined.

| ***Parameters*** | ***Interval  (sensitivity)*** |
| --- | --- |
| Map size | [30 – 100] |
| Number of steps in the simulation | [700 – 1300] |
| Detection radius | 1 |
| Inertia in pollinators’ random movement | [0.01 – 0.4] |
| Number of flowers (as function of meadow area) | [0.02 – 2] |
| Coefficient of nectar refill | [0.001 – 0.005] |
| Use of repellent scent mark | True or False |
| Time spent probing each flower | [1 – 10] |
| Coefficient of scent mark disappearance | - coeff_nectar |
| Pollinators per flower | [0.1 – 2] |

Our analysis explained over 95% of the variance, and the relative importance of the parameters is highly variable (Fig S2.1). Our results suggest that the coefficient of nectar refill is the parameter that most influences the amount of nectar collected. Consequently, we decided to examine three values in our study: 0.001, 0.0025, and 0.005, meaning that the flower can refill at most one, two, or five times during the simulation. The second parameter with a high impact on the amount of nectar collected is the number of pollinators per flower, which is a major determinant of the intensity of competition and one of the most important aspects of this study.

The random search movement depended on the *sigma* parameter, a modelling choice that result, in very rare occasion, on a change of travel angles superior to 2π radians. Nevertheless, this parameter was the one with the lower sensitivity, having little impact on our response variables.


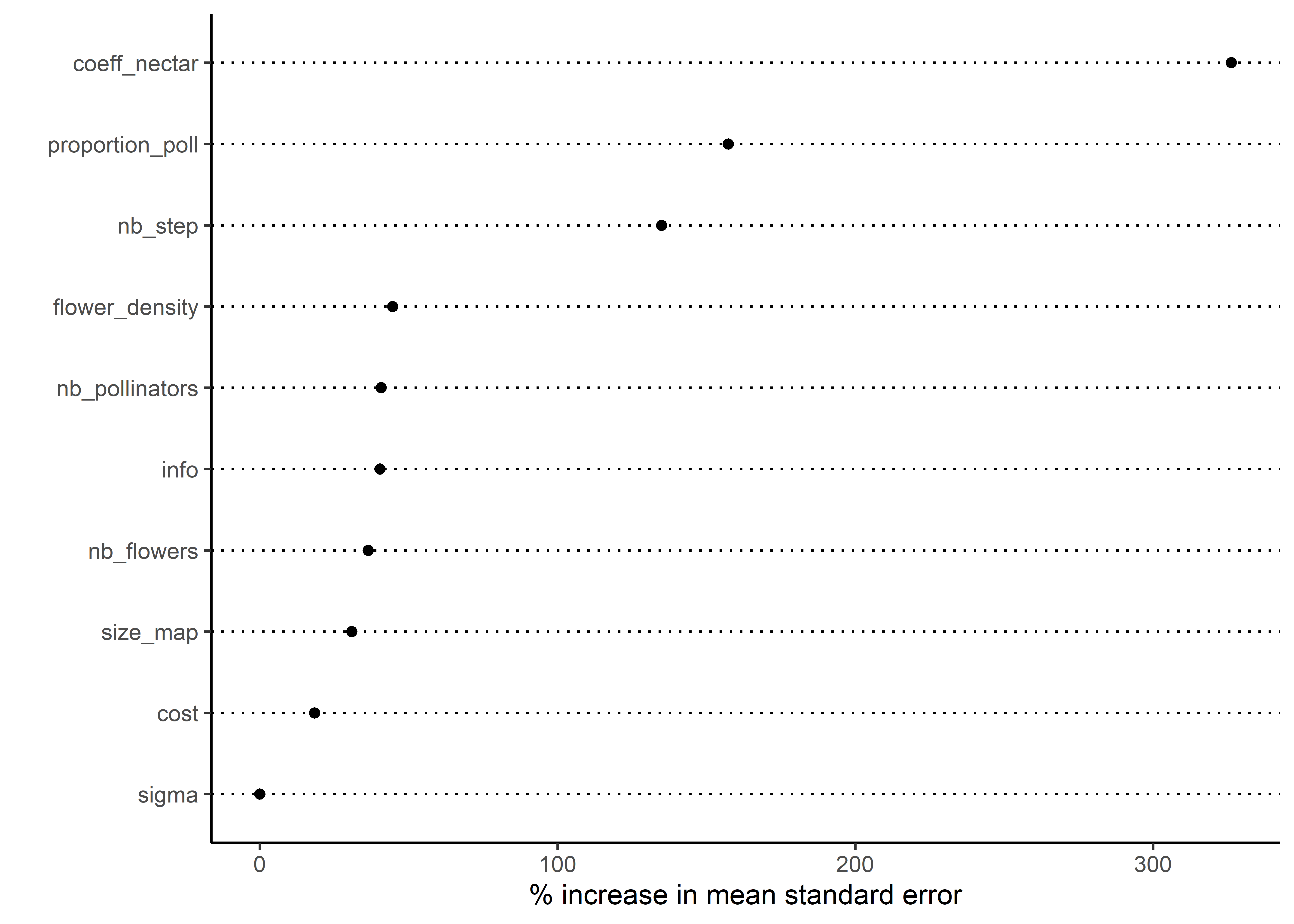


Fig S2.1: Importance of parameters on the variation of the mean nectar quantity collected by pollinators, defined by a random forest analysis.

The number of steps has a large impact on the amount of nectar collected, because it gives the pollinators more or less time to collect. This variable influences the duration of the simulation, and to keep a reasonable simulation duration, the parameters were set to 1,000 steps. The flower density, the number of flowers and pollinators, and the size of the map increase the mean standard error of the model by almost 50%. All these parameters are linked to the intensity of competition. Nonetheless, the number of pollinators influences the duration of the simulation (see Fig S2.2). To keep a reasonable simulation duration, we decided to set the maximal number of pollinators to 500, the density of flowers and the size of the map were set in consequence. The choice to use public information will also affect the amount of nectar collected, which is interesting for our problem.


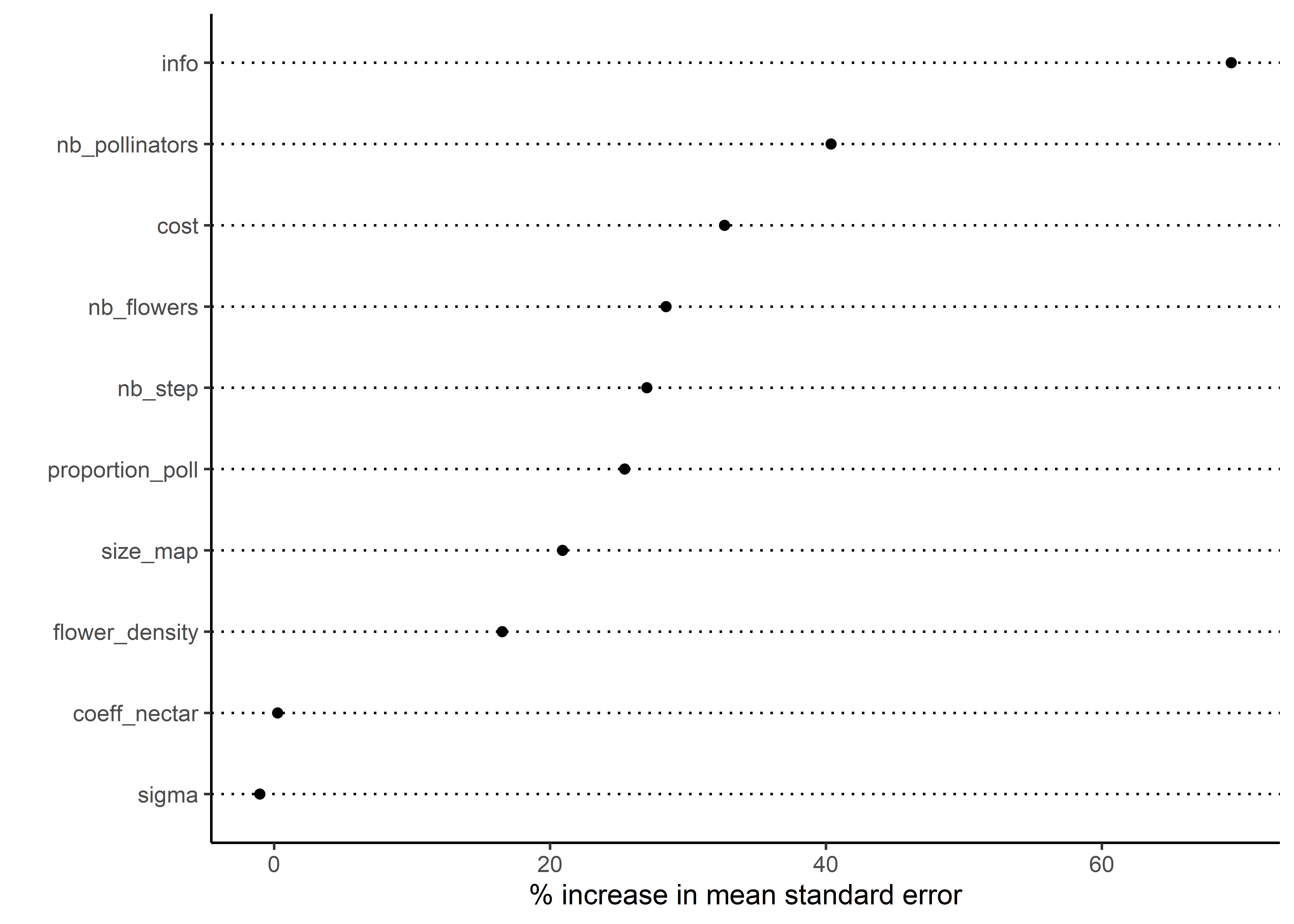


Fig S2.2: Importance of parameters on the variation of the simulation’s duration defined by a random forest analysis.

References

1. R Core Team. R: A language and environment for statistical computing [Internet]. Vienna, Austria; 2020. Available from: https://www.R-project.org/

2. Liaw A, Wiener M. Classification and regression by randomForest. R News. 2002;2(3): 18-22.
