# Supplementary material for: Modelling the effects of the repellent scent marks of pollinators on their foraging efficiency and the plant-pollinator community
Source: PLoS One. 2021 Sep 8;16(9):e0256929. doi: 10.1371/journal.pone.0256929 (PMC8425561; doi:10.1371/journal.pone.0256929)
Supplement: S3 File — Coefficient of variation of the number of visits per flower during the simulation and the Coefficient of variation of the number of flowers which receive a visit of a pollinator after he leaves the focal one. (DOCX) [file pone.0256929.s003.docx]

S3 File – Extra Figures





*Fig S3.1: (A) Coefficient of variation of the number of visits per flower during the simulation. (B) Coefficient of variation of the number of flowers which receive a visit of a pollinator after he leaves the focal one. In each figure, the x-axis represents the relative abundance of pollinators compared to flowers.*

On the Fig S3.1A, we can see that the number of visits per flower is less variable when the abundance of pollinators increases and when they use the scent mark.

The Fig S3.1B, shows that when the pollinators do not use the scent-mark, the pollen is more equally disperse among the flower community.
