# Supplementary material for: Modelling the effects of the repellent scent marks of pollinators on their foraging efficiency and the plant-pollinator community
Source: PLoS One. 2021 Sep 8;16(9):e0256929. doi: 10.1371/journal.pone.0256929 (PMC8425561; doi:10.1371/journal.pone.0256929)
Supplement: Attachment — Submitted filename: PLOS-ONE_review.pdf [file pone.0256929.s004.pdf]

Review Comments

Overview:

In their manuscript, the authors explore the use of scent marks left on the corolla of flowers by pollinators, as a cue to avoid recently depleted resources. They use a spatially explicit agent-based model to examine the effects at the individual and collective level. They show that, at the individual level, the use of scent marks increases foraging efficiency (increases nectar collection) when competition for resource is important. At the community level, this resulted in a homogenization of the resource depleted. Their results also show that the use of scent marks decreased the number of flower visits, which can potentially reduce the pollination services.

The manuscript is written clearly and the results are intuitive and appear to be in line with empirical data. My main concern is about their conclusion saying that the use of scent marks can potentially decrease pollination services.

Major comments:

1. In their results, the authors show that the use of scent marks reduces the number of flower visits. They mention many times that this reduction in flower visits implies a reduction of pollination services. This implication is not necessarily true, especially because their results also show that the use of scent marks homogenizes the visits among flowers. In line 307, the authors cite [40] which indicates that the visitation rate is related to pollen receipt. We can understand that pollen receipt is related to pollination services although no reference is provided here. However, the distribution of the pollen among flowers and not only the total amount is important. To put an example, it is not clear to me that a landscape consisting of 5 flowers with 100 visits to one of the flowers and 0 visits to all the others, has more pollination services than a landscape in which each of the 5 flowers get 10 visits. In the first case, the total number of visits is 100, in the second case is 50, but I expect the second case to have higher pollination services. This example is very related to their case of study and so their conclusion “fewer flowers visits imply less pollination” isn’t generally valid. The decrease of flower visits when using scent marks supports the reduction of pollination services, but the decrease of the coefficient of variation of the number of visits per flower when using scent marks supports the increase of pollination services. For that reason, the authors should provide a better justification for their conclusion or change it. To evaluate the effects of scent marks on pollination services another metric should be used. I suggest plotting the number of flowers with no visits, or the number of flowers with less than “x” visits as a possible indicator, although I think a conclusive argument would be hard to achieve.
2. The authors do not specify with enough detail the type of movement their model bees do. In Fig.2, the sentence “Move of one arbitrary unit in almost the same direction as previously” is too vague. What does an arbitrary unit mean? What does almost the same direction mean?
3. The authors do not mention previous modelling work about scent marks. For example, there is quite a bit made by Mark Lewis and other authors in papers such: Brian K Briscoe, Mark A Lewis, and Stephen E Parrish. Home range formation in wolves due to scent marking. Bulletin of Mathematical Biology, 64(2):261–284, 2002.

Minor Comments:

Text:

1. 49 Replace “their consumption” with “the consumption”
2. 51 Can you include some examples illustrating what is meant by “personal information”? Does personal information also indicate memory?
3. 85-89. The sentences “We aim to … nectar available on the flower” says the same as the next lines “we record … and mean number of visits per flower”, it sounds repetitive.
4. 164 Section number “2.3” is repeated.

Plots:

1. Use the same notation on the vertical axes in Figs. 3, 4 and 5 when possible. For example, in Fig. 3, plot A has on the axis “Nectar collected”, so plot B should therefore be “Relative nectar collected”. In Fig. 4, plot A has on the axis “Nectar remaining” (is it per flower? if so, then put it, if it is not, then don’t put per flower in plot B, since in B is relative abundance, it doesn’t matter if it is per flower or not). In Fig. 5, plot A says “Nectar remaining”, plot B says “Relative abundance of nectar per flower” shouldn’t it be simply “Relative nectar remaining”?
2. There is no dotted red line in Fig. 3 as mentioned in the caption of the figure.
3. Make a legend for Figs. 3, 4 and 5. It is much more visual than going to the caption every time to see which colour corresponds to the use of scent marks and which not. The legend should also include information about the nectar replenishment rates represented by “squares”, “triangles” and “circles”. I also encourage you to use a different colour than “red” (only for scent mark pollinators) and “blue” (only for no-scent mark pollinators) for the case of relative abundances.

Suggestions:

1. I suggest adding a future direction considering the memory of scent marks (so foragers would do flights more directly to their favourite locations in the landscape) or scent mark dependent dynamics (foragers moving faster when they perceive high concentrations of scent marks around them). These types of behaviours can potentially increase the efficiency of foragers using scent marks even more.
2. If your model can reproduce the behaviour stated in lines 272-274, why you don’t add something in the abstract? It seems relevant to me.
